# Editorial: Digital remote patient monitoring in neurodegenerative diseases

**DOI:** 10.3389/fdgth.2025.1725819

**Published:** 2025-11-11

**Authors:** Amit Khanna, Diane Stephenson, Graham Jones

**Affiliations:** 1 Novartis AG, Basel, Switzerland; 2 Critical Path Institute, Tucson, AZ, United States; 3 Tufts Medical Center, Boston, MA, United States

**Keywords:** remote monitoring, sensors, disease progression, composite digital biomarkers, speech, ocular, regulatory pathways, real world evidence

Remote monitoring is gaining prominence in patient care enabling the recording of repeat measurements outside of scheduled clinical visits. In neurodegenerative diseases this is of added significance as changes that take place are often subtle, span multiple domains (motor, cognition, sleep, speech, etc.) and new symptoms develop over several years. This special volume integrates perspectives from experts and thought leaders in the academy, pharmaceutical industry, and research foundations who highlight significant new developments.

One of the important elements for adoption of remote monitoring devices in clinical studies is patient acceptance and to adhere over sustained periods necessary to derive clinically meaningful data. In a review of wearable device adoption rates, Hirczy et al. conducted online surveys to identify barriers to uptake among Parkinson's disease patients. Surprisingly, among US based patients although greater than 90% of respondents were interested in new technologies only 24% were using consumer devices for disease management and only 8% with medical grade wearables.

Similarly, Kangarloo et al. report patient experiences with body-worn sensors used in clinic and a mobile application used at-home from the WATCH-PD study. This observational, 12 month study focused on disease progression in early Parkinson's Disease among 82 participants with PD and 50 control participants. Results demonstrated that participants had generally positive views on comfort and use of the technologies throughout the study duration regardless of group. Significantly, device proficiency and acceptability in people with early stage PD did not differ from neurologically healthy older adults, providing impetus for future clinical studies.

Careful study design is paramount when implementing new technologies in clinical settings including assessing the reliability of the data captured. Lavine et al. examine the test-retest reliability of accelerometry derived data from at-home studies. Using raw data derived from triaxial accelerometry involving 21 PD patients and 23 controls they applied linear mixed models to determine the identity of drug treatment effects. They conclude that at-home measures have favorable reliability profiles as more data points can be gathered, and the reduction in sample size needed to detect progression presents clear justification for their deployment in future studies.

The design of long term studies of disease tracking will likely require development of innovative computational approaches to data capture and interpretation. Zhai et al. present a new machine learning framework to construct composite digital biomarkers for progression tracking. The framework was applied to data collected from an observational PD study involving movement measurements captured using the Opal TM sensor combined with MDS-UPDRS Part III scores. The composite digital measure exhibited a smoother and more significant increasing trend over time with less variability, and ability to classify between *de novo* PD and healthy controls.

Although a majority of studies have focused on movement and motion tracking, there are a number of exciting developments on the horizon with alternative measures. Speech and acoustic signals are a potentially very rich source of clinical information in neurological diseases and Tröger et al. highlight recent findings on speech intelligibility. They describe a digital measure for speech intelligibility which was deployed on datasets from patients suffering from Dysarthria, a motor speech disorder associated with Parkinson's Disease (PD), progressive supranuclear palsy (PSP), Huntington's Disease (HD) and amyotrophic lateral sclerosis (ALS). The score, derived from automatic speech recognition (ASR) systems, showed good to excellent inter-rater reliability and significant differences in intelligibility scores between pathological groups and healthy controls.

Ocular analysis is another area of promise and Band et al. provide a timely overview of the study of eye movement abnormalities to indicate neurological condition severity and distinguish disease phenotypes. Recent strides in imaging sensors and computational power have resulted in a surge in the development of technologies facilitating the extraction and analysis of eye movements to assess neurodegenerative diseases. Their review provides an overview of these advancements, their potential to offer patient-friendly assessments and explores current trends and future directions in this exciting field.

Other approaches are being developed with the similar goal of detecting diseased states at population level using low patient burden technologies. Jiang et al. reflect on studies in Canada where automated facial expression analysis (AFEA) was compared to standard measures such as electroencephalogram (EEG) technologies and heart rate variability (HRV). The case for development of composite measures of cognitive decline based on AFEA is presented, and its utility in remote deployment using contactless data capture supported by potential economic benefits through the national healthcare system.

Advancing digital remote monitoring technologies for drug development studies requires careful approach to study design and ultimately alignment with prevailing regulatory guidance. In a timely overview the role of the Critical Path Institute is highlighted, bridging key interfaces between the health authorities, pharmaceutical industry sponsors, patient advocates, and the clinical research community Stephenson et al. Progress made through the Critical Path for Parkinson's Consortium's (CPP) Digital Drug Development tool (3DT) serves to showcase their approach. The initiative has helped accelerate the regulatory maturity of several key digital health technology measures, and advanced thinking on approaches to clinical trial design, data acquisition and the use of AI methodologies to extract critical features.

A tenet in regulatory guidance for remote patient assessment is the need to focus on activities of daily living (ADL) and real world evidence. An emerging trend for patient monitoring is the development of smart home environments, with sensors and devices located strategically to capture key health related data. Grammatikopoulou et al. report findings on the assessment of ADLs in subjects at the CERTH-IT simulated Smart Home. Sensor data was used to track activity as subjects (controls and groups suffering from cognitive decline) conducted various tasks and operations. Differentiation between controls and other groups was attainable and valuable feedback obtained to refine the approach for wider deployment.

These are exciting times for the deployment of patient monitoring technologies in neurodegenerative diseases. Progress highlighted by these leaders is having demonstrable impact on moving the field forward. We hope this inspires others to innovate, challenge hypotheses, and develop practical solutions to advance new treatment options and ultimately influence patient care. Clearly, there is more to come.

Sincerely,

Amit Khanna, Diane Stephenson, Graham Jones

